# Medical and entomological malarial interventions, a comparison and synergy of two control measures using a Ross/Macdonald model variant and *openmalaria* simulation

**DOI:** 10.1016/j.mbs.2018.04.005

**Published:** 2018-06

**Authors:** R.C. Elliott, D.L. Smith, D. Echodu

**Affiliations:** a Micron School of Materials Science and Engineering, Boise State University, Engineering Building Suite 338, Boise, ID 83725, USA; b Pilgrim Africa, 115 N 85th St #202, Seattle, WA 98103, USA; c Institute of Health Metrics and Evaluation, University of Washington, 2301 Fifth Ave., Suite 600, Seattle, WA 98121, USA

**Keywords:** Malaria, Indoor residual spraying, Mass drug administration, Ross/Macdonald, Openmalaria, Simulation

## Abstract

Using an established Ross/Macdonald model variant for mosquito-born parasite transmission, we extend the formalism to simply incorporate time-dependent control measures. In particular, two interventions are considered, mass drug administration (MDA) and indoor residual spraying (IRS), whose individual intensities during their respective campaigns are set to the same intervention-reduced reproductive number *R*_0_. The impacts of these interventions, measured as each campaign’s ability over time to reduce infections in a community, are found based on the transmission setting, coverage, and their associated durations. These impacts are compared for both interventions and their joint deployment. Synchronous campaigns of IRS deployed with MDA have a cooperative, synergistic effect whose impact exceeds that when the campaigns are deployed in isolation. Simulations with *openmalaria*, with its more complex model of transmission, are separately performed and show a similar impact enhancement with these interventions. A new, associated analysis yields simple scaling relationships that estimate the dynamical resurgence time, post-intervention, to infection proliferation in a community.

## Highlights

• An adaptation of the classical Ross–Macdonald model for vector disease transmission to incorporate time-dependent medical and entomological control measures.
• Modeling both mass drug administration and indoor residual spraying campaigns, the synchronous deployment of both yields a synergy where the impact of a joint intervention exceeds that of isolated campaigns.
• Openmalaria simulations, separately run, indicate comparable intervention impacts to the Ross/Macdonald model variant.
• The vector reservoir of parasitemia is found to be labile, and this dictates the impacts of the medical and entomological interventions.
• A scaling-law level of analysis is performed that estimates the rebound of infections in a community after interventions expire, and not only do higher transmission environments bounce back to prevalent infections faster, communities with stronger interventions are shown to have a slower relapse to parasitemia.

## 1 Introduction

Modeling and simulation are eminently appropriate tools for optimizing malaria control strategies [1], helping elucidate their probable mechanisms of action, and optimizing intervention timing and coordination. To compare different methods of suppressing infection in a community with endemic disease, we extend an established Ross/Macdonald model variant for mosquito-born parasite transmission to simply incorporate time-dependent control interventions. In particular, we focus on mass drug administration (MDA) and indoor residual spraying (IRS), two strongly effective interventions which address, respectively, the human and the mosquito reservoirs of parasitemia. These interventions have direct and complementary effects on the transmission dynamics.

There is context and precedence for this investigation from a long history of both MDA and IRS campaigns, sometimes used in combination. In the post-WWII era, DDT and Chloroquine prompted large scale campaigns with the ensuing Global Malaria Eradication Programme of 1955–1969 [2], [3]. Subsequent eradication efforts both succeeded and failed [4], [5] and a legacy of this era is acquired resistance [6]. Both Chloroquine and DDT lost efficacy with successive sprayings and the regularly high chemical pressure stemming from frequent treatment with a single pharmaceutical. Later, from 1969 to 1976, the Garki project investigated and recorded many aspects of transmission, immunology, entomology and epidemiology [7] and both IRS and MDA campaigns were carried out as isolated interventions and in tandem. When both IRS and MDA interventions were applied in combination, the regular, high coverage (85%) campaigns were able to achieve a high level of control, though unable to fully interrupt transmission [8]. Such control, though it does not progress directly to elimination, will be relevant for the modeling of these interventions below, as we investigate the mechanism for achieving this pre-elimination level of suppression. Beyond Garki, many MDA [9], [10], [11] and IRS campaigns [12], [13] have been carried out with varying successes, and recently reviewed for Africa [14], [15].

We consider the isolated and combined impact of MDA and IRS campaigns in a few different transmission settings. Recent modeling efforts have investigated similar control interventions and combinations of them [16], [17], [18], [19]. Generally, the control measures are carried out as finite programs that are deployed, have a prescribed duration, and promptly end. Post-campaign, in the absence of further interventions or a permanent alteration of the entomological environment, modeled communities eventually rebound to their previous transmission balance where malarial infections are again prevalent. As such, eradication scenarios are not directly considered below but rather the process of large-scale reductions in the host and vector reservoirs is, and this is an important component of any elimination program.

The Ross/Macdonald model with control interventions, below, allows MDA and IRS campaigns to be generalized and understood in an illuminating manner, revealing a significant synergistic effect between them, which, if robust, has potential utility in malaria control policy. The aim of the application of this Ross/Macdonald variant is not so much to emphasize detail or force some precision in its extension, but to look for general trends and to elucidate the dynamics of parasite transfer between the host and vector reservoirs, especially when it is restricted with control interventions. As a result, the modeling is relatively crude and has few parameters. In fact, the IRS intervention below, other than prescribing a coverage and duration, is a *single-parameter* intervention. This sole parameter is also easily interpreted: it essentially benchmarks the (reduced) reproductive number $R_{0}^{I}$ during the intervention. This adapted and extended formalism is relatively easy to use and understand, but correspondingly, has somewhat limited flexibility and muscle. It is best employed, as here, for robust features and trends.

To ensure that the observed synergistic effect and its size are not a quirk of the simple semi-analytical model, we have also run *openmalaria* simulations. The *openmalaria* simulations incorporate tremendous detail absent in the simple Ross/Macdonald model, including acquired immunity, demographic information included for transmission intensities and a health system for case management, and so afford a meaningful comparison with the much simpler dynamical model. Interventions are simulated as closely as possible with those described in the text in the semi-analytic Ross/Macdonald model. However this comparison is not perfect. Our intention is not to make an absolutely complete, direct comparison on directly equal footing between the semi-analytic model and the full malarial transmission model of *openmalaria*, but to see if some simple features are in correspondence, and potentially also find contrast, if it exists.

We also establish a novel scaling result that details a community’s collective response, post-intervention, to re-infection. Malaria will invade again when campaigns expire and this timeline of re-establishing parasitemia depends on the transmission intensity and other entomological factors, though it is complicated by many forces wholly absent in a “clean” modeling environment [20]. Estimated from a stability analysis in an accompanying appendix, this rate of re-infection is referenced throughout. The rate enables a general, effective period of intervention efficacy: the waiting period prior to the need for new control measures. To our knowledge, it has not been established in the literature.

## 2 Methods: A dynamical variant of the classical model

We begin with a simple population model of local mosquitoes harboring parasites and capable of infecting hosts. Using classical notation, the proportion of this mosquito population is *Z*, and for simplicity will here consist of a single, short-lived anopheles vector with an average lifespan of $g^{-1}$. This is the sporozoite rate and it grows as more mosquitoes become infectious, acquiring parasites through bloodmeals with infectious hosts, and wanes with their death. Among the very simplest dynamical descriptions of the sporozoite rate is (with others reviewed in [21]) Smith and McKenzie [22],

(1) $$\frac{dZ}{dt}=acX\left(e^{-gn}-Z\right)-gZ,$$

which on the right hand side consists of creation and annihilation terms for these rates. The second is clearly the force of mortality, which is here the only means of diminishing the infectious population. The first creates more infectious mosquitoes through biting events with infectious hosts. If the vector bites hosts periodically at an average rate of *a* (or the feeding time interval is $a^{-1}$), and the proportion of infectious humans is *X*, infectious mosquitoes are created at a rate of *acX*, where *c* is a human-to-mosquito transmission efficiency. New infectious mosquitoes are then created at a per-mosquito rate of acX(1−Z), essentially the first term in Eq. (1). An extrinsic incubation period for infectiousness *n* is incorporated here by diminishing the eligible infected vector population so that only exp(−gn) of newly infected anopheles survive to become infectious, a limitation based on the simple hazard model for mosquito survival. Incorporating the delay in this simple fashion avoids the distinction of *infected* and *infectious* populations of mosquitoes, and the need to monitor them separately. Mosquitoes below are thus considered as infectious or not, with the creation rate of the infectious fraction given by $acX(e^{-gn}-Z)$. Mosquitoes are assumed to carry infectiousness, once acquired, to their death.

The evolution of the infectious proportion of humans *X* in a transmission setting is similarly constructed. First, a host recovers, on average, from a malarial infection at a rate of *r*, and is only infectious $r^{-1}$ days post-initiation. The infectious host population evolves similarly to the above sporozoite rate, and is written [23],

(2) $$\frac{dX}{dt}=mabZ\left(1-X\right)-rX.$$

The first term creates the infectious populace, again through coupled biting events. The rate that uninfected humans (1−X) become infected is *mabZ*, where *a* is again the rate of biting events, and *b* is the efficiency coefficient for mosquito-to-human transmission [24]. This term is augmented by the prefactor *m*, which is the population ratio of mosquitoes to humans (and can be very large in high-transmission environments). This first term, mabZ(1−X) is constructed as the creation rate of new, infectious human hosts from the naive host populace and is ignorant of the time lag of parasite development in the hosts. Ignoring this latency, the infectious population of humans is again exactly equal to that of the infected proportion of humans. A summary of the seven classical parameters of transmission is given in Table 1.

**Table 1:** The Ross/Macdonald classic transmission parameters and their *four* elemental combinations to characterize dynamics.

(a) The classic Ross/Macdonald parameters and their units.		(b)The basic four unitless transmission parameters.	
m	Ratio of mosquitoes to humans	$R_{0}=\frac{ma^{2}bce^{-gn}}{rg}$	The basic Reproductive number
a	Host feeding rate (bites per mosquito per unit time)	γ=ac/g	Number of bites in a mosquito’s
b	Transmission efficiency, infectious host to mosquito		lifetime that infects it
c	Transmission efficiency, infectious mosquito to host	β=mab/g	Number of infecting bites per
$g^{-1}$	Average mosquito lifetime (time)		host in a mosquito lifetime
n	extrinsic incubation period (time)	$P_{e}=e^{-gn}$	Probability a mosquito
$r^{-1}$	Host infectious period (time)		survives incubation latency

These dynamics neglect a large number of complicating influences in both the entomology and epidemiology. First, obviously the spatial component to transmission [25], [26], [27], and the fact that both mosquito and host populations are moving ones is absent [28], [29], [30] and is something that will not be discussed here further. Also, host immunity, access to care, and super-infection are wholly neglected, and seem to be culpable, for instance, in one poor performance of Ross/Macdonald models in a high-transmission setting [31]. Simple Ross/Macdonald dynamics also overlook any heterogeneities in host selection, the role of parasite densities in transmission events, in-host dynamics and a more proper inclusion of innoculation latencies. A review of models with these embellishments can be found in reference [32]. Due to these complications, the regime of validity for either one of expressions, Eqs. (1) and (2), is generally not known nor discussed. It is reasonable to suppose, however, that low-to-mid intensity transmission environments, those considered below, might be most amenable to these simple dynamics; infectious transactions may be assumed to be prevalent, and complications such as super-infection play less of a complicating role. Seasonal entomological trends are also a strong dynamical influence and are similarly discarded here in order to focus on the effects of interventions in a very simple setting.

Eqs. (1)and (2) describe a dynamical overview of the transmissive elements for infection and provide perhaps the *simplest* tranmsmission model, for host and vector dynamics. Infectious proportions are boosted with coupled density transmission events and diminished with vector death or the expiry of host infections; these are the only means in which infectiousness is gained or lost.

### 2.1 Analysis: Basic dynamics and stability.

Analyzing the system Eqs. (1)–(2) for fixed points, there are two, as is well-known [33], [34]. The first is the trivial solution, X=0 and Z=0, which is an elimination scenario, with neither infectious mosquitoes nor hosts present; there are no parasites transmitted in the host/vector system. The second is a dynamical equilibrium with infectious parasites present in both human hosts and mosquitoes,

(3) $$X^{*}=\frac{R_{0}-1}{R_{0}+\gamma }\;\;\;\text{and,}\;\;\;Z^{*}=\left(\frac{\gamma P_{e}}{R_{0}}\right)\frac{R_{0}-1}{1+\gamma }.$$

Two definitions, γ=ac/g and $P_{e}=e^{-gn}$ have been used and the reproductive number *R*_0_ has been introduced,

(4) $$R_{0}=\frac{ma^{2}bce^{-gn}}{rg}=me^{-gn}\left(c\frac{a}{g}\right)\left(b\frac{a}{g}\right)\left(\frac{g}{r}\right).$$

The reproductive number, the number of infected humans created from a single infected host in an otherwise naive population [23], [35], is the product of the mismatched ratio of populations *m*, the mosquito’s probability of surviving the extrinsic incubation period $P_{e}=\exp\left(-gn\right),$ contributions from the two bites necessary for infection transmission, *ac*/*g* and *ba*/*g*, and a factor which is the ratio of the amount of time an average mosquito has with an infectious host, *g*/*r*. The product of the middle terms is *a*^2^*bc*/*g*, an absolute count of the (lifetime average) successful number of two-fold transmissive events taking place per mosquito. Prefactors diminish or augment this simple count: the mosquito population in *m* has more mosquitoes (linearly) causing more overall transmission, and their mortality during incubation *P_e_* diminishes it. The reproductive number *R*_0_ alone quantifies transmission intensity, a result of the balance of the creation and loss terms of Eqs. (1) and (2).

Rewriting the system above with two transformations, first dividing the infectious populations by their (nontrivial) stable points, $\bar{X}=X/X^{*}$ and $\bar{Z}=Z/Z^{*},$ and second, scaling time by the mosquito lifetime τ=gt. The system of Eqs. (1) and (2), transforms to,

(5) $$\begin{array}{ccc} \frac{d\bar{X}}{d\tau } & = & \beta \left(\frac{Z^{*}}{X^{*}}\right)\bar{Z}\left(1-X^{*}\bar{X}\right)-\alpha \bar{X} \end{array}$$

(6) $$\begin{array}{ccc} \frac{d\bar{Z}}{d\tau } & = & \gamma \left(\frac{X^{*}}{Z^{*}}\right)\bar{X}\left(P_{e}-Z^{*}\bar{Z}\right)-\bar{Z}, \end{array}$$

with the following redefined parameters: α=r/g is the mosquito lifespan divided by the human infectious period (as mentioned above, this is the ratio of time an average mosquito has with the human infectious period), β=mab/g is the lifetime average number of successful infecting bites per host, and γ=ac/g, as above, is the number of bites in a mosquito’s lifetime that infects it. It is clear with Eqs. (5)–(6) that *γ* mediates human-to-mosquito interactions while *β* mediates mosquito-to-human ones. The reproductive number is also simply expressed with the scaled parameters, $R_{0}=\gamma \beta P_{e}/\alpha ,$ which emphasizes its alternative interpretation which is that it is the ratio of two rates, that of infections invading the community *γβP_e_*, divided by the rate they leave, *α*. The resulting dynamical system in Eqs. (5)–(6) (and thus Eqs. (1) and (2)) is thus fully specified by just *four* independent parameters: *R*_0_ and *γ* specify X* via Eq. (3), adding *P_e_* specifies Z*, and finally *α* (*β*) yields *β* (*α*). The complete set is {*R*_0_, *γ, β, P_e_*}, consisting of parameters of the transmission intensity of the setting *R*_0_, *γ* and *β* are scaled interaction parameters for mosquito-to-human mediated transmission and vice-versa, and lastly $P_{e}=\exp\left(-gn\right)$ is the proportion of mosquitoes that survive the incubation period. The incubation period *n*, as in Eq. (1) is an obvious alternate specification. Table 1 summarizes the seven classical transmission parameters and the four elemental ones.

The conditions of stability and some other dynamical considerations for the system of Eqs. (5) and (6) are analyzed in Appendix A. In brief, stability about the elimination point, $\bar{X}=\bar{Z}=0$ is lost when conditions merit *R*_0_ > 1, or when the community has infection rate that outpaces its recovery. All trajectories for reproductive numbers *R*_0_ < 1 attract to the elimination point, $\bar{X}=\bar{Z}=0$ and all others attract asymptotically to the stable, non-trivial equilibrium point {X*, Z*}, that of Eq. (3). We mention these domains explicitly, though they are widely understood, only because the control interventions considered below will adjust the reproductive number, if temporarily. With good coverage and effective chemoprevention and/or vector control, the reproductive number can fall below unity for the duration of the intervention (high effect size [36]). In this period, dynamics exist on a trajectory that attracts towards elimination. Elimination will however not result from these measures if retraction or intervention expiry causes only a temporary reduction in transmission, and these are the cases explored below. A rebounding *R*_0_ re-introduces transmission and results in a dynamical relaxation to the equilibrium of Eq. (3), re-establishing malaria in the community.

## 3 Theory: The addition of interventions

In this section, interventions are modeled as control efforts which change the reproductive number, *R*_0_, through (say) enhanced mosquito mortality or diminished human infectious periods. These approximated effects perturb the evolution of the infectious populations subject to estimated impacts. For example, periods of intervention activity/duration are approximated, and dynamical trajectories are calculated through the interventions, via Eqs. (5) and (6), but with time periods of suppressed $R_{0}^{I}<R_{0}$ and associated parameters. Upon intervention expiry, the system dynamically relaxes according to the same ambient conditions prior to the intervention. Noted above, and in more detail in Appendix A, globally the system can relax to only one of two fixed points: the trivial equilibrium of elimination, $X^{*}=Z^{*}=0,$ or the stable transmissive environment given by Eq. (3) where parasites are exchanged freely and there are measurable populations of infectious hosts and mosquitoes at modest *R*_0_ > 1. This relaxation—the slow (or fast) acquisition of malaria in the community, post-intervention—including the characteristic times of regaining equilibrium, is also explored below.

Again, the modeling of MDA and IRS interventions is intentionally simplified. Our interest is to investigate general trends and extract robust, scaling-level effects. For this purpose, three time windows are isolated for the evolution of the infectious proportions *X*(*τ*) and *Z*(*τ*), which are before, during and after the intervention period. The dynamics evolve according to transmission intensity of each time-period: $R_{0},R_{0}^{I}$ and *R*_0_ respectively (superscript *I* denotes an intervention-period value), with the bottlenecked dynamics of the intervention explored in more detail below. Prior to the intervention, the trajectory is static, and that of a community stuck at the stable equilibrium points of Eq. (3). All evolving dynamics are stable with freely exchanged parasites from hosts to mosquitoes and vice-versa. Upon the deployment of either MDA or IRS (or both) campaigns, the second time period, a proportion of the host/vector populations is affected by the intervention, and transmission is immediately reduced. This means, for example, when an MDA is deployed and the coverage is 85% of a local population is treated,  ∼ 85% of the infectious pool of humans has their parasite load cleared and their infectious status is exactly voided. This value of the infectious proportion, $\bar{X}\left(\tau =\tau_{0}\right),$ with *τ*_0_ a time of intervention deployment, is then used as an initial condition for the subsequent dynamics of the intervention period, with an associated set of parameters appropriate to the intervention: $\left\{R_{0}^{I},\gamma^{I},\beta^{I},P_{e}^{I}\right\}$. The time duration of this second period is estimated, which for an MDA is based on the duration of prophylaxis offered by the particular drug administered. Upon expiry of the MDA, the dynamics return to those of the time period prior to the intervention, again with the original parameters, {*R*_0_, *γ, β, P_e_*}. The evolved densities $\bar{X}\left(\tau_{0}+\bar{\tau }\right)$ and $Z({\bar{\tau }}_{0}+\bar{\tau })$ at the expiry of the intervention period with duration $\bar{\tau }$ are used as initial conditions for post-intervention system evolution. This is the starting point for the system’s third window, to dynamically relax to one of the two fixed points post-intervention: elimination, or proliferation and the return to an infected populace. In the below, since the pre-elimination parameter values are instantaneously switched back on after $\tau =\tau_{0}+\bar{\tau }$ (for example *R*_0_ is once again suddenly large), elimination can only be achieved with exactly $X(\tau_{0}+\bar{\tau })=0$ (*never*): the elimination fixed point is unstable for *R*_0_ > 1.

It should be mentioned with these simplifications that decaying intervention effects are not present here. There is no decaying efficacy of an insecticide for example; interventions are taken to be working or wholly ineffective. We choose not to parameterize individual insecticides or medical interventions, but only prescribe an initial impact and effective duration. As will be seen, these generalizations not only enable a simple analysis, they illuminate some of the forces responsible for an intervention’s impact, and in particular engender an understanding of the interactions between multiple interventions.

All non-analytic and non-simulation results are full solutions to the coupled nonlinear Eqs. (5) and (6), integrated numerically with a fourth-order Runge–Kutta algorithm as the system itself is not (at least not easily) separably integrable. Parameter values are indicated in the text or figure captions.

### 3.1 Mass drug administration

The effects of an MDA campaign are incorporated through estimates of coverage and duration, as well as adjustments to two internal parameters which account for the altered transmission during the campaign’s effective period. First, infections, or here, infectiousness, is squelched at the time of administration and $\bar{X}\left(\tau_{0}\right)$ is set to just 15% of its equilibrium value, a number which corresponds loosely to the campaign’s coverage, but also necessarily involves a chosen drug’s efficacy, and compliance and/or adherence to its prescriptive treatment. Second, the duration of the MDA also depends on the selection of administered drugs (any parasite resistance is here clearly disregarded), and for simplicity the period of the MDA is taken to be $\bar{\tau }=2,$ or two mosquito lifetimes (maybe three weeks with $g^{-1}\approx 10$). Lastly, systematic effects of the MDA are also ascribed through two internal parameters, *r* and *b*, discussed below. Adjustments to these two parameters change the dynamical course of Eq. (2) (or Eq. (5)) through changes in the coefficients, but the prefactors of Eqs. (1) and (6) remain as before. The MDA does not change the entomology—*Z*(*τ*_0_) remains unchanged by the MDA—at the deployment time of the MDA, and the sporozoite rate responds only after this moment. The local infectious proportion of anopheles changes with the MDA only through the coupled dynamics of the infectious populations.

As host infections are cleared for the recipients of the MDA, the average duration of the human infectious period, $r^{-1}$ (contained in α=r/g above), decreases as a result. Those infections cleared have shorter durations and, on average, the host infectious period reduces. Transmission mosquito-to-human is also correspondingly squelched, a chemoprophylactic effect for treated individuals, so *b* (in *β* above) also diminishes. Through these two modifications, the reproductive number, $R_{0}=\gamma \beta P_{e}/\alpha$ plummets. To accomplish these reductions, the host healing rate is amplified, *r* → *ξr* (or *α* → *ξα* in the above) with *ξ* > 1. This rate amplification diminishes the average duration of a host infection, an obvious effect of administered anti-malarials. The mosquito-to-human transmission for the short period of the campaign is also greatly reduced, a prophylactic effect, and *b* → *b*/*μ* (β=mab/g→β/μ) is set to accomplish this. As a consequence, the reproductive number is squelched *R*_0_ → *R*_0_/*μξ* for the short duration of the intervention, corresponding to an effect size of $R_{0}/R_{0}^{I}=\mu \xi$ [18], [36], [37]. This reduction is not entirely independent of the coverage, or vice-versa, in that if coverage is weak, the reduction in *R*_0_ must correspondingly be modest. We focus here on relatively high impact interventions and high coverage in an effort to focus on relatively potent interventions and what may be achieved with them.

Just after the effective time period of the MDA, these effects promptly expire and the parameters return to their pre-intervention values. Succinctly, the MDA campaign has the following settings: an initial impact related to the intervention coverage *X*(*τ*_0_), the duration of the MDA effective period $\bar{\tau }=2,$ the reduction in the human infectiousness period $r^{-1}\to {\left(\xi r\right)}^{-1},$ and the chemoprophylactic effect with *b* → *b*/*μ*. The reproductive number $R_{0}^{I}=R_{0}/\xi \mu$ follows from these, again, only during the short time window of the campaign.

With these parameter simplifications, infectious host dynamics during the MDA period $\tau_{0}<\tau <\tau_{0}+2g^{-1}$ are determined from,

(7) $$\frac{d\bar{X}}{d\tau }=\frac{\beta }{\mu }\left(\frac{Z^{*}}{X^{*}}\right)\bar{Z}\left(1-X^{*}\bar{X}\right)-\xi \alpha \bar{X},$$

and the intervention values of these prefactors are $\alpha^{I}=\xi \alpha$ and $\beta^{I}=\beta /\mu$. The sporozoite rate evolves as before, according to Eq. (6) as both *γ* and *P_e_* are unaffected by the intervention: the medical intervention alters only host dynamics directly. The sporozoite rate changes only “downstream,” through preempted parasite transmission. It is worth noting that the proportions in Eq. (7) are still normalized by their pre-intervention equilibrium values, X* and Z*.

After the MDA initially causes human infectiousness to plummet with the clearing of the host reservoir, $\bar{X}\left(\tau_{0}\right)=0.15,$ subsequent host dynamics are determined from the evolution of Eq. (7) with *ξ* and *μ* set with two conditions. The first is that these rescalings reduce the reproductive number $R_{0}^{I}=R_{0}/\xi \mu ,$ so *ξ* and *μ* must be set so that $R_{0}^{I}<1,$ as host infectiousness should diminish and attract towards the elimination point $X^{*}=0$ during a campaign that is effectively administered with robust anti-malarials. The second condition is to roughly balance the terms on the right hand side of Eq. (7). This selection is made as a sensible estimate: an MDA generally maintains the clearing of the host reservoir during its effective period. That is, if 85% of the population is properly treated with an anti-malarial that has a prophylactic period post-administration, we might expect that host infectiousness $\bar{X}$ will (at least) not increase, but also hold relatively steady during the period of effectiveness of the drug. If, on the other hand, a particular drug does not have a robust prophylactic effect, *μ* can be modified (reduced) to accomplish this. Values for *ξ* and *μ*, regardless of *R*_0_, in the cases below are always set with $R_{0}^{I}=0.5,$ which reflects a very effective campaign. The effect size varies among different ambient transmission settings set with *R*_0_, meaning that here $R_{0}^{I}=0.5$ is maintained regardless of the initial, pre-intervention *R*_0_.

**Fig. 1:**
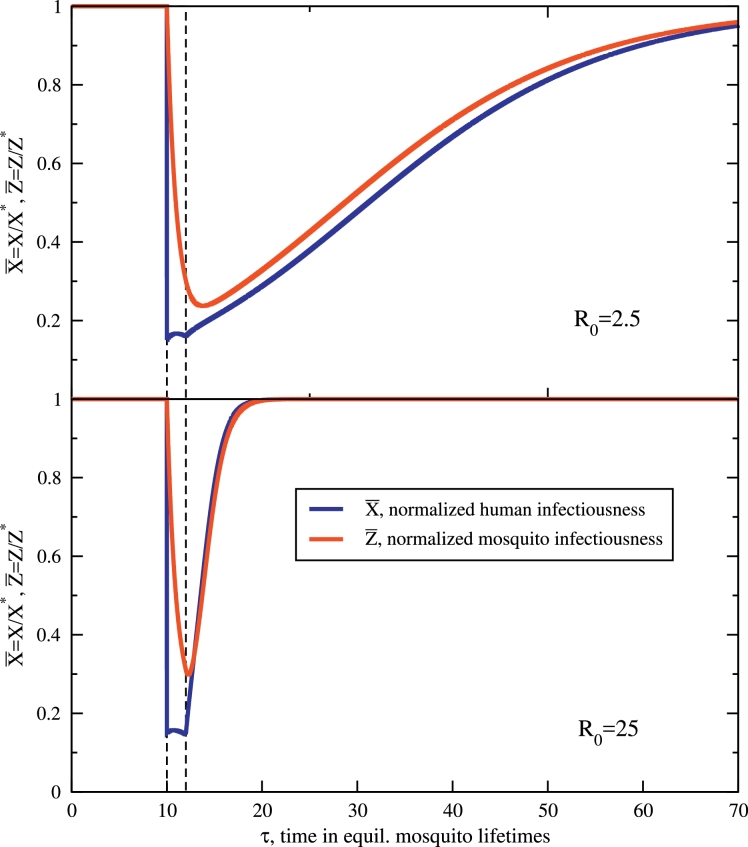
Normalized trajectories for human $\bar{X},$ and mosquito $\bar{Z}$ infectiousness with an applied, model MDA at (nondimensional) time τ=10. The application period is highlighted in both panels. The top figure shows the long-lasting effects of an MDA campaign in a low-transmission ($R_{0}=2.5$) environment, which still has effects tens of mosquito lifetimes later. Below, the same MDA has a much shorter effect, recovering in a few mosquito lifetimes for a moderate transmission environment, $R_{0}=25$. The average human infectious period is diminished by an introduced prefactor ξ=3 during the campaign and *μ* is set preserving $R_{0}^{I}=0.5$ during the period of the applied MDA. These two parameters are introduced in the text and dictate the chemoprophylactic effect of the administered anti-malarial (*μ*) and its associated ability to clear average host infectiousness (*ξ*). The other parameters are specified in correspondence with previous work [29], [38], [39], which are γ=0.642,$\alpha ={\left(150d\right)}^{-1}/{\left(10d\right)}^{-1},$ and $P_{e}=1/e$. More analysis and details of the MDA are in the text.

The integrated dynamical trajectories of the MDA campaign are shown in Fig. 1, for a low ($R_{0}=2.5$) and moderate ($R_{0}=25$) transmission setting. The host and mosquito prevalence curves are normalized by their pre-intervention equilibrium values of Eq. (3), so they both initiate and, upon expiry of the intervention, bounce back towards equilibrium at $\bar{X}\to 1,\bar{Z}\to 1$. It is clear that during the period of the MDA campaign, $\tau_{0}<\tau <\tau_{0}+\bar{\tau },$ with the diminished reproductive number, $R_{0}^{I}<1,$ dynamics for both infectious densities decay towards the elimination stable point (albeit somewhat slowly by construction for $\bar{X}$). The initial impact of an 85% reduction in $\bar{X}$ is striking, but the sporozoite rate also drops with alacrity, *i.e.* the vector parasite reservoir *depletes quickly*, in just a few mosquito lifetimes. The sporozoite rate is clearly very labile with respect to changes in the host infectious density. The MDA campaign targets and mostly clears a very large reservoir of parasites in hosts. The infectious portion of the more ephemeral mosquito population adjusts quickly to reflect these changes. At the end of the MDA campaign, for $\tau >\tau_{0}+\bar{\tau }=12,$ the intervention has expired and host protection is promptly lost. Mosquitoes continue to bite hosts and individuals previously receiving treatment and prophylaxis are eligible to become infected once again. The stable equilibrium of freely exchanged parasites with X* and Z* in Eq. (3) is regained in time. This decaying approach to X*, Z*, prompted by these uninfected individuals from the MDA becoming re-infected takes much more time in the low-transmission $R_{0}=2.5$ setting than the high-transmision one, a relatively obvious result, but one which supports the use of MDA in the context of elimination. A stability analysis, described in more detail in Appendix A, estimates the characteristic recovery time(in units of $g^{-1}$) of the infected host population to be,

(8) $$\tau_{+}\approx \frac{1+\gamma }{\alpha R_{0}}=\left(\frac{1}{\beta P_{e}}\right)\frac{1+\gamma }{\gamma },$$

so that large *R*_0_ clearly bounces back faster, as is seen in Fig. 1. This recovery time, a non-trivial function of transmission parameters, is a decreasing function of *γ*, as well as *β* and *P_e_*. Boosting the human-to-mosquito (or mosquito-to-human) transmission coefficients decreases the time to regain equilibrium. The relation in Eq. (8) is also somewhat unique in that post-campaign, *α* is conspicuously absent: only those fast dynamics mediated by the entomology and host-transmission, *γ, β* and *P_e_*, are relevant (at first order in *R*_0_). As such, while the medical treatment of an MDA campaign clearly affects the recovery time of treated individuals (information contained in *α*), it is the entomology and parameters of transmission, absent these in-host effects, that causes the community as a whole to lapse once again into a state with prevalent malarial infection. The use of pharmaceuticals for the in-host clearance of infections can establish impressive gains, but the forces for invading parasitemia post-prophylaxis are all first-order dependent on transmission from the vector, despite its depleted reservoir. This foreshadows the needed role for vector control that will be considered in the next sections.

Post-intervention, the rate malarial infections invade the populace may also be written,

(9) $$\lambda_{+}=\frac{1}{\tau_{+}}=\lim_{X\to 1}g^{-1}f_{I}\left(X\right),$$

with

(10) $$f_{I}\left(X\right)=\frac{ma^{2}bcXe^{-gn}}{g+acX}=g\frac{\beta P_{e}\gamma X}{1+\gamma X}.$$

Here *f_I_*(*X*) is the force of infection, the rate of successful infectious bites received per human. Here, as classically, it is a function of the infected host proportion. A comparable estimate (note expression (8) is approximate) is,

(11) $$g^{-1}f_{I}\left(X^{*}\right)=\alpha \frac{R_{0}-1}{1+\gamma }\approx \frac{\alpha R_{0}}{1+\gamma }=\lambda_{+}.$$

The restoration rate, the rate malarial infections return to the community, is essentially the force of infection of the pre-intervention entomological setting. It is the rate of planted, viable infectious bites, that of the entomological setting into which the system relaxes.

Before moving on, it is important to remark on Eq. (8) (or Eqs. (9) and (11)) as a scaling relation. It is derived in the linearized approximation of the Eqs. (5) and (6), and as such has relevance in the neighborhood of fixed points such as X*, Z*, while neglecting the nonlinear coupling in these regions. For this matter, the MDA campaign above upon expiry is not a small perturbation from the fixed point of Eq. (3), and the scaling estimate for recovery post-intervention is relatively numerically poor here. A better estimate is found with some further analysis in Appendix A. It is,

(12) $$\tau_{m}=gf_{I}^{-1}\log\left(\frac{1+{\bar{X}}_{f}}{{\bar{X}}_{f}}\right)=k_{f}\tau_{+},$$

where ${\bar{X}}_{f}=\bar{X}\left(\tau_{0}+\bar{\tau }\right)$ is the infectious host proportion at intervention expiry. This recovery time is dependent on the suppressed host infectious proportion ${\bar{X}}_{f}$ at the end of the intervention: the more parasitemia is suppressed from the campaign, the longer the period of restoration, a sensible result. For this matter, it takes longer for the malarial infections to return in a community where a more successful MDA campaign has taken place. This effect amounts to essentially enhancing the recovery time to a few multiples $k_{f}\equiv \log\left(1+{\bar{X}}_{f}^{-1}\right)$ of the characteristic time $\tau_{+}$ (Eq. (8)), $\tau_{m}=k_{f}\tau_{+}$ above.

Despite these rates and recovery times being valid for a linearized approximation, the response time of Eq. (8) (or (12)) is certainly not without merit given that it illuminates the entomology and transmission properties that are responsible for restoring equilibrium, which is especially true as *X*(*τ*) approaches X* ($\bar{X}\to 1$). Also, perhaps the most conspicuous attribute of Fig. 1 is the difference in recovery times for the two transmission settings post-intervention. The scaling of this rebound in Eq. (8) with ${R_{0}}^{-1}$ indicates the relative recoveries of X* differs by an order of magnitude, which matches the recovery trends in the figure extremely well: one is almost exactly ten times longer than the other. One final interesting property explored in Appendix A is that the relapse time of Eq. (8) does not endlessly shorten with *R*_0_ but *saturates*, ultimately achieving the asymptote $\tau_{+}\approx 1+\gamma ,$ another consequence of the fast turnover of the vector population.

Of course, in a real setting the force of infection does not instantaneously revert back to pre-intervention levels, as has also been assumed here, but more likely continuously regains momentum as the effect of an intervention deteriorates. This waning influence of an intervention should delay, or slow, reemergence from this estimate. Since immunity is additionally absent in this analysis and it potentially protects some individuals from re-infection, slowing the influx of parasitemia, this estimate is also likely fast from this oversight. Eq. (12) can thus be regarded as a fast estimate, or short threshold, for the characteristic resurgence time.

### 3.2 Indoor residual spraying

An IRS campaign changes the local, ambient vector population and *inter alia* the reproductive number. Contrasting the MDA campaign, IRS does not change human infectiousness with deployment, so $\bar{X}\left(\tau_{0}\right)$ remains unchanged at this point; IRS affects human infectiousness dynamics only “downstream,” as fewer infectious mosquitoes survive to bite humans and transmit infections.

To account for the killing effects of an IRS campaign, a skeletal population model of mosquitoes is required. Killing anopheles with insecticide is an effect contained in *m*, the ratio of populations, mosquitoes to humans. This ratio *m*, embedded in the composite parameter *β*, has thus far been regarded as constant, but for the next few paragraphs alone, we regard it as changing in time to account for the insecticidal killing effect (it will be shown that this time-dependence is spurious). Among the simplest of population models [21], mosquitoes have an emergence/mortality balance,

(13) $$\frac{dm}{dt}=\epsilon -gm,$$

with ϵ a per-host emergence rate and *g* the force of mortality as above. In correspondence with the treatment for MDA, all terms are normalized by an unchanging human population of *N, i.e.* m=M/N, with *M* the total relevant mosquito population: those proximal, female, and host-seeking. The equilibrium population ratio is $m^{*}=\epsilon /g,$ a balance of birth and death rates. The population dynamics of Eq. (13) has some deficiencies (one emphasized below), but is nonetheless capable of generating insight into the main drivers of transmission reduction. According to this population model, if an IRS campaign diminishes the population to *m*_0_ from the equilibrium value of m* at $\tau =\tau_{0},$ the mosquito population recovers as,

(14) $$m\left(\tau \right)=m^{*}+\left(m_{0}-m^{*}\right)e^{-\left(\tau -\tau_{0}\right)}.$$

Note that the characteristic time of recovery is (of course) $g^{-1},$ the average mosquito lifetime (again, τ=gt, as above and all time is scaled with the equilibrium mosquito lifecycle). Constant mosquito emergence replaces the lost population in a matter of just a few mosquito lifetimes. This highlights one inherent weakness of the emergence/mortality balance of Eq. (13): the emergence rate is undeterred despite a reduced population of mosquitoes. If perhaps an application of insecticide kills a substantial number of adult mosquitoes, their (likely) fewer offspring do not result. For the same reason, as designed, vector extinction is similarly impossible. Shortcomings such as these will be overlooked in the following in favor of a coarse look at vector control that simply maintains a lower population of mosquitoes when active.

Since IRS kills mosquitoes, with *m* falling with its deployment, the mosquito mortality rate *g* must increase. To capture this, for the effective length of time of the IRS campaign, *i*.e. roughly the viable period of the insecticide, the mortality rate is boosted, *g* → *κg*, where *κ* > 1 here augments the force of mortality and correspondingly diminishes the average lifetime of anopheles. The reproductive number *R*_0_ thus decreases with both fewer mosquitoes *m* and their shortened lifecycle, diminishing transmission. Augmenting the force of mortality in Eq. (13), with *g* → *κg*,

(15) $$\begin{array}{ccc} m(\tau ) & = & \frac{m^{*}}{\kappa }+\left(m_{0}-\frac{m^{*}}{\kappa }\right)e^{-\kappa \left(\tau -\tau_{0}\right)} \end{array}$$

(16) $$\begin{array}{c} \;\;\;\;\;\;\approx \frac{m^{*}}{\kappa },\;\;\text{for}\;\;\tau -\tau_{0}>\kappa^{-1}, \end{array}$$

the mosquito population re-equilibrates to m*/*κ*, reducing the equilibration above by a factor of *κ*. The recovery time, that to regain the new equilibrium m*/*κ* is short, changing from $g^{-1}$ to ${\left(\kappa g\right)}^{-1},$ so that not only does the population rebound to a reduced equilibrium, it does so quickly. Because the population recovery time is shorter than an average mosquito lifetime, the second term in Eq. (15) is a fast transient effect, and $m=m^{*}/\kappa$ alone is sufficient to capture the population reduction for all but the very shortest (irrelevant) time scales. In the following, *m* no longer contains this time-dependence but during an IRS campaign their ambient population is reduced by the augmented mortality rate *m* → *m*/*κ*.

The rescaling of *g* during the IRS adjusts the mosquito-to-human transmission prefactor, β=mab/g as well as the fraction of mosquitoes that survive the incubation period, $P_{e}=e^{-gn}\to e^{-\kappa gn}$. With the same scale normalization as before, the evolution equations for the IRS transformations are,

(17) $$\begin{array}{ccc} \frac{d\bar{X}}{d\tau } & = & \frac{\beta }{\kappa }\left(\frac{Z^{*}}{X^{*}}\right)\bar{Z}\left(1-X^{*}\bar{X}\right)-\alpha \bar{X} \end{array}$$

(18) $$\begin{array}{ccc} \frac{d\bar{Z}}{d\tau } & = & \gamma \left(\frac{X^{*}}{Z^{*}}\right)\bar{X}\left({P_{e}}^{\kappa }-Z^{*}\bar{Z}\right)-\kappa \bar{Z}, \end{array}$$

and note the time τ=gt is still measured in units of $g^{-1}$ and not ${\left(\kappa g\right)}^{-1}$ with this construction. The IRS evolution is modified with a boosted mortality rate *g* → *κg*, an altered mosquito-to-human transmission prefactor *β*, and a diminished survival during the incubation period, *P_e_* → *P_e_*^*κ*^. Fewer survive to pass on parasites with this amplified mortality. Other than the initial reduction and the campaign duration common to all interventions, it should be noted that the IRS evolution is configured with only a single parameter, *κ*, which changes these factors. It can be shown the reproductive number is reduced by,

(19) $$R_{0}=\frac{\gamma \beta P_{e}}{\alpha }\Rightarrow R_{0}^{I}=R_{0}\frac{{\left[P_{e}\right]}^{\kappa -1}}{\kappa^{2}},$$

a scaling with *κ* that has been pointed out previously [22].

A last detail is needed for the IRS, which is the connection between a campaign’s coverage and how the sporozoite rate adjusts with its deployment, setting $\bar{Z}\left(\tau_{0}\right)$. This is a rather subtle point, and we might expect that the reduction in the number of ambient mosquitoes *m* → *m*/*κ* be adequate for a description of the insecticide’s effect: mosquitoes would be killed indiscriminately and the fraction harboring infections to be unaffected. This is however not the case because the boosted mortality rate *g* → *κg* of the campaign modifies the age structure of mosquitoes, and infectious mosquitoes are by requirement older, having survived the latency period. To this end, the hazard model for mosquito survivorship has an age distribution given by exp(−gt)≡exp(−τ), with average lifetime $g^{-1}$. The percentage of the total that could be infectious (they are old enough to have survived the extrinsic incubation period) is $P_{e}=\exp\left(-gn\right)$. During the IRS campaign, the age distribution modifies to exp(−κτ), with shorter average lifetime ${\left(\kappa g\right)}^{-1},$ and the proportion of mosquitoes who are old enough to be infectious reduces to exp(−κgn). Thus, the ratio of those that live long enough to be infectious, post-IRS to pre-IRS, is ${P_{e}}^{\kappa -1}=\exp\left(-gn\left(\kappa -1\right)\right),$ and is the fraction affected by a perfect (coverage of unity) IRS campaign. If an IRS campaign affects a fraction of this mosquito population with coverage *c*_0_, the total reduction at deployment is, $\bar{Z}\left(\tau_{0}\right)=\left(1-c_{0}\right)+c_{0}{P_{e}}^{\kappa -1}$. With $R_{0}=25,$ the reduction in $\bar{Z}\left(\tau_{0}\right)$ is 71% at deployment for 85% coverage, a substantial reduction.

**Fig. 2:**
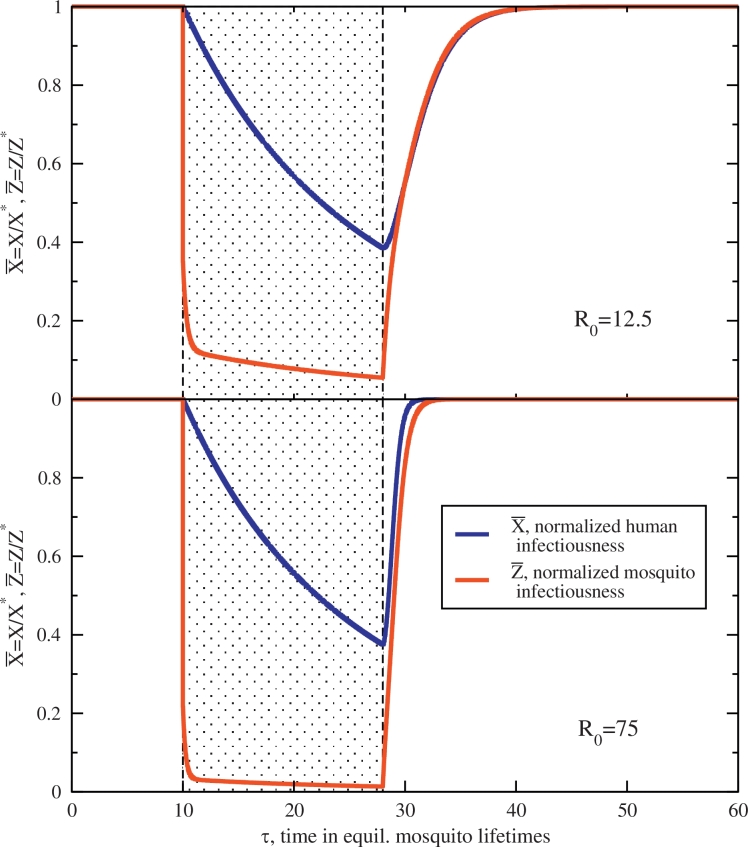
Normalized trajectories for human $\bar{X}\left(\tau \right),$ and mosquito $\bar{Z}\left(\tau \right)$ infectiousness with an applied, model IRS at time τ=10. The application period, which has duration $\bar{\tau }=18$ (18 mosquito lifetimes, maybe a bit more than a half year) is highlighted in both panels. The top figure shows the long-lasting effects of IRS in a low-transmission ($R_{0}=12.5$) environment, which recovers slowly post-intervention. Below, the same IRS has a similar effect, but recovers rapidly post-intervention in the medium-high transmission environment, $R_{0}=75$. Mosquito lifetimes are diminished by an introduced prefactor *κ* set to preserve $R_{0}^{I}=0.5$ during the intervention. Other parameters are set as before and more details are in the text.

The effects of this model IRS campaign for two transmission settings are shown in Fig. 2 with 85% coverage, synonymous to the initial reduction in $\bar{X}$ for the MDA. The intervention duration is much longer for the IRS than the immediate cleansing of an MDA campaign, as insecticide is generally viable for months, and set here to $\bar{\tau }=18$ (or $18g^{-1}\approx 180$d, maybe half a year), a time window highlighted in the figure. There is no decline in the efficacy in the insecticide: it is either on and working during the $18g^{-1}$ period of the IRS, or exactly off. Comparable to the MDA above, the augmentation of the mosquito mortality rate is again set to preserve $R_{0}^{I}=0.5$ for all transmission environments (which through Eq. (19) sets *κ*). It is reasonable to expect the sporozoite rate to decline during the effective period of the IRS yielding a net killing effect, diminishing $\bar{Z}$ and attracting to the stable node of elimination, $Z^{*}=0$. Upon retraction, however, prevalent infectious densities again return. Expiry of the IRS here causes the community to be subject to re-infection from the fast dynamics of the recovering vector parasite reservoir. $\bar{Z}$ quickly bounces back towards the proliferation stable point and host infectiousness follows, scaling with the entomology, the ambient force of infection, as before in Eq. (12).

Another important comparison with an MDA campaign illustrated here is that the host infectiousness $\bar{X}$ is *slow* and even comparatively *sluggish* to respond to changes in the sporozoite rate $\bar{Z}$. This is in direct contrast with the MDA campaign above, where fast changes in $\bar{X}$ result in fast changes in $\bar{Z}$. The reverse is not true. Fig. 2 indicates a slow decay in host infectiousness after the mass killing of infectious mosquitoes during the IRS. In fact, the sporozoite rate plummets to roughly a tenth of its equilibrium value for nearly half a year, and host infectiousness decays consistently and comparably slowly. This asymmetry is a result of infections lost only through expiry at a rate of *r* (see Eq. (2)), a rate directly modified by the MDA but untouched by the IRS. It is only the long duration of the IRS that allows host infections to clear and $\bar{X}$ to wane. The IRS period has only a trickle of new infections due to a strongly suppressed mosquito population, but with no mechanism or program in place to actually clear existing host infections, waiting out the infectious period is the only means to a reduced presence of parasitemia in the community. The clearing of host infections in an MDA results in a correspondingly fast clearing of the vector reservoir, but the cleansing of the vector reservoir with an IRS does *not* result in the fast clearing of the host reservoir. Host infections expire only at rate *r* (contained in *α*), *i.e.* slow.

A final note on the model IRS campaign here regards the domain of applicability of the intervention. With the labile nature of the sporozoite rate, low transmission environments *R*_0_ ≲ 10 (not shown in Fig. 2) present a situation where high host infection $\bar{X}\approx 1,$ and suppressed $\bar{Z}$ post-deployment, do not balance the creation and destruction terms of Eq. (1). In these low-transmission environments, anopheles infection can still outpace the relatively (and necessarily) modest *κ* reduction responsible for the accelerated killing of mosquitoes for a short time. As a consequence, for small pre-intervention *R*_0_ there is a transient period immediately post-IRS deployment where the sporozoite rate grows (responding quickly to the yet-still high $\bar{X}$) but then subsequently falls, as it must attract to the elimation fixed point $\bar{Z}=0$ with $R_{0}^{I}<1$ by construction. This takes place in a short time post-deployment where briefly the creation term outpaces annihilation. As configured, the reproductive number $R_{0}^{I}=0.5$ is here preserved absolutely, regardless of initial *R*_0_, which may be a tremendous reduction in high-transmission settings but a fairly modest effect in low transmission (the effect size varies). Preserving this constraint in low-transmission settings, chosen in part to coordinate a comparison with the MDA at the same $R_{0}^{I}=0.5,$ has as a consequence a poor balance of terms in Eq. (18) during the intervention. For this reason, somewhat larger *R*_0_ values are depicted in Fig. 2 than Fig. 1, avoiding these transients at low *R*_0_.

## 4 Results

### 4.1 Synchronous IRS and MDA, modeled by the Ross/Macdonald variant

**Fig. 3:**
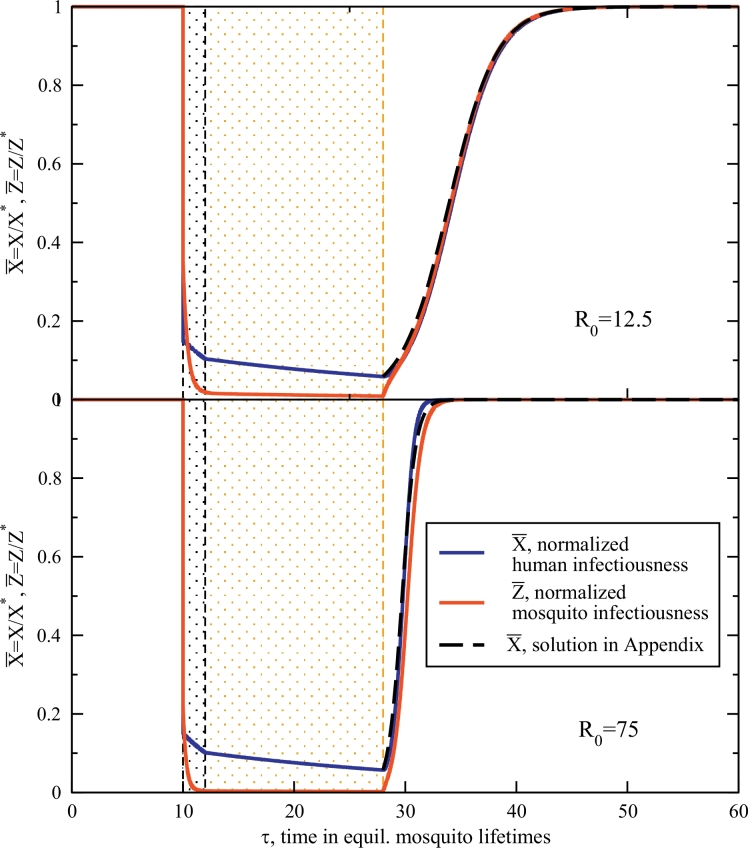
Normalized trajectories for host $\bar{X},$ and mosquito $\bar{Z}$ infectiousness when both an MDA and IRS are deployed synchronously at τ=10. All intervention standards are as before. The MDA clears infectiousness in 85% of the populace for a duration of $\bar{\tau }=2$ (2 mosquito lifetimes), with this time window highlighted in the figures. The IRS reduces the sporozoite rate by 85% at the same time and is effective for a duration of $\bar{\tau }=18,$ as before, and also highlighted in the figures. A low transmission figure again has a slow recovery (top, with $R_{0}=12.5$) and the higher transmission setting bounces back quickly at the expiry of the effective IRS time-span, at τ=28. A weakly nonlinear solution explored in Appendix A is also drawn.

Trajectories for a synchronous deployment of an IRS and MDA are plotted in Fig. 3. Synchronous deployment has the infectious host proportion depleted to just 15% and the sporozoite rate rather comparably reduced from their pre-intervention equilibrium values, in correspondence with the individual campaigns considered above. The interventions are deployed exactly as described in Section 3, with the same durations and reduction parameters. Here, the MDA campaign is finished at τ=12, and dynamics are then determined by the IRS evolution equations, Eqs. (17) and (18), as it continues beyond the MDA period to τ=28. Intervention durations are indicated on the figure as before. Comparable to the cases above, at the expiry of the IRS, the parameters instantaneously revert to their environmental settings for transmission prior to the campaigns. Fig. 3 shows this rebound for both host and vector prevalence post-campaign. The lower transmission setting regains equilibrium in the long time limit while the high transmission setting rebounds quickly, again in agreement with the scaling result of Eq. (12). Also plotted are integrable solutions found by exploiting the fact that the sporozoite rate responds very quickly to changes in the host infectiousness. Appendix A has more details on these trajectories.

The most important feature of Fig. 3 is that during the period of the campaign, prevalence is low, nearly in the pre-elimination regime, even for the high-transmission setting. Host infectiousness/infections are first cleared by the MDA to low levels (compare with Fig. 1), and rather than this depletion of the host reservoir being short-lived, this level of prevalence is maintained by the diminished force of infection accomplished by the IRS. In short, the gains of the MDA are sustained by the IRS.

**Fig. 4:**
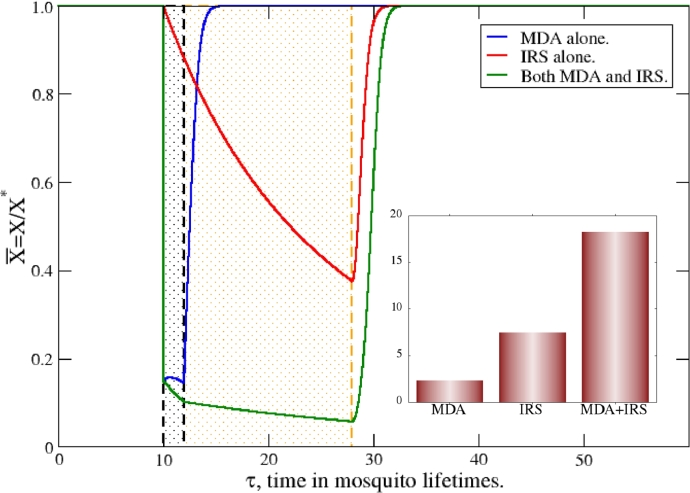
Direct comparison of trajectories for human $\bar{X}$ infectiousness when MDA and IRS are deployed individually and together, in a medium-high transmission setting, $R_{0}=75$. All intervention standards are as before. Suppression from synchronous deployment clearly exceeds that of either IRS or MDA alone, and the inset demonstrates this synergy. The inset shows the impacts (Eq. (20)) of the interventions, indicating the nearly twofold enhancement of the combination of synchronously deployed MDA+IRS. This is a *synergy* of the interventions.

These gains in reduction with combined campaigns are notable because the infection suppression is better than additive. A direct comparison for the high transmission setting, $R_{0}=75,$ is shown in Fig. 4. Host infectiousness trajectories for isolated MDA and IRS campaigns, deployed individually, are compared with that of the combined, synchronous IRS/MDA. It is clear the combined deployment has a *synergistic* effect, clearing far more infections than either alone.

To measure the robustness of a campaign or campaigns, and to compare effective interventions or intervention sequences, we define their impact, deployed individually or together, as,

(20) $$I=\int \left[{\bar{X}}_{0}\left(\tau \right)-{\bar{X}}_{c}\left(\tau \right)\right]d\tau .$$

Here ${\bar{X}}_{0}\left(\tau \right)$ and ${\bar{X}}_{c}\left(\tau \right)$ are the prevalence trajectories for the community without interventions and with them, respectively. ${\bar{X}}_{c}\left(\tau \right)$ with subscript *c* denotes a trajectory with included campaigns. As defined, the impact clearly simplifies for the Ross/Macdonald variant, with ${\bar{X}}_{0}\left(\tau \right)=1,$ since the prevalence absent interventions is simply the average value of the transmission setting. It is written more generally here as it will be applied for simulation cases, below, where stochastic noise is present and ${\bar{X}}_{0}\left(\tau \right)\neq 1,$ but fluctuates about unity. The impact is simply the area in Fig. 4 bound by the trajectories with campaigns, ${\bar{X}}_{c}\left(\tau \right)$ and those without, ${\bar{X}}_{0}\left(\tau \right),$ and is simply interpreted as the percentage of infections prevented by the campaign times its effective time. As such, a short duration campaign that deeply cleanses the host reservoir, such as a high coverage MDA in a high transmission setting, may have a net impact *I* comparable to a different, low coverage intervention with a correspondingly long duration. The impact measures the total abilities of the intervention (or a sequence of several) to stem infection.

On this note, the integration limits for Eq. (20) must be large enough to encompass all effects of the intervention(s), especially if their duration is substantial. Provided the time window begins prior to the time of the intervention and is suitably large for long term effects, its duration does not matter. Times *τ* far in advance or long after the campaign have ${\bar{X}}_{c}\left(\tau \right)\approx {\bar{X}}_{0}\left(\tau \right)$ and do not contribute; the impact *I* is constant over a (suitably large) window of integration. There are however a few subtleties for simulations, which are mentioned in Appendix B.

The impacts for an isolated MDA, isolated IRS, and the synchronous MDA+IRS are shown in the inset to Fig. 4, and demonstrate the enhanced suppression of combined, jointly-administered interventions. The impact of the synchronous MDA+IRS is *roughly double* the impact of an isolated MDA with that of an isolated IRS campaign. This is particularly noteworthy because campaigns that are scheduled and deployed strategically, *i.e.* jointly administered, vastly outperform two deployed separately, temporally in isolation. This is to say roughly twice the control can be accomplished by jointly deploying the interventions.

### 4.2 Interventions and their impacts with *openmalaria* simulation

Individual-based simulations are separately run with *openmalaria* as a comparison for these transmission settings and interventions, and especially to test for the robust synergy in Fig. 4. These simulations include significant detail compared to the semi-analytic model presented above and common ground for comparison is difficult. Several features of *openmalaria* that are wholly absent in the above model include demographic heterogeneity of transmission, partial immunity of the population, case management in health systems, and variable transmission/infectivity based on a list of factors [40], [41], [42], [43], [44], [45], [46], [47]. The entomology and vector lifecycles are also separably configurable. An appendix below (Appendix B) details several aspects of the simulations run here, and we focus on a comparison that does not necessarily try to strip down *openmalaria* and compare the base models of transmission and interventions, but rather highlight how reasonably comparable cases yield the same trends. For example, in the below, case management is not entirely absent (though it is minimal), and acquired partial immunity is included in most results, though clearly neither of these are contained in the Ross/Macdonald model above. However, since these additions are integral components of the *openmalaria* model and to some degree, an elaboration of simpler, underlying transmission models, the intention below is not to force a comparison of exact situations but to see if the more sophisticated simulation approach may also yield a comparable signature of intervention impact and their timings. As will be seen, adhering to general cases and incorporating the most essential features of the interventions enables a reasonable correspondence between the simulation and semi-analytic model.

**Fig. 5:**
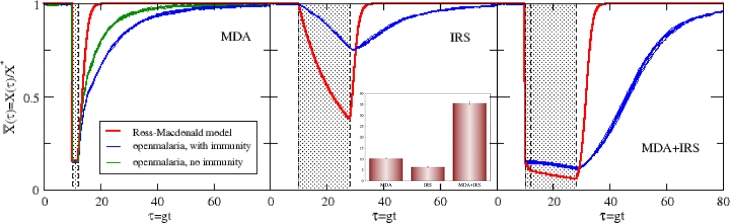
Normalized, infected host trajectories for *openmalaria* simulations and the semi-analytic Ross/Macdonald model in the text, in three intervention scenarios. For the simulations, these are the normalized proportions of hosts with any parasite density ( ≥ 0.01 parasites/µ*L*). In the left panel of the tryptic, two sets of *openmalaria* trajectories are displayed, with and without immunity. Eight trajectories for these simulation sets are shown in all cases to demonstrate the implicit stochastic noise. The left panel shows the impact of an MDA campaign deployed at τ=10, as in cases before. The middle panel shows the impact of an isolated IRS campaign, and the right shows synchronously deployed campaigns. Effective periods of the interventions are highlighted in all panels. The annual EIR is set to $E_{a}=25$ bites/annum, for $R_{0}=25,$ a correspondence from ref. [48] and further discussed in Appendix B. The inset shows the corresponding impacts (Eq. (20)) for these three cases, demonstrating the synergy of synchronous deployment. The small error bars on this inset encompass the stochastic variance in *I* for all simulated trajectories.

Fig. 5 shows the three situations described above, an isolated MDA, an isolated IRS, and the synchronous deployment of both. Plotted in the tryptic are the Ross/Macdonald semi-analytic theory presented above, and two sets of simulation trajectories for $R_{0}=25$ (corresponding to an annual EIR of $E_{a}=25$ bites/host*annum with no seasonal variation, see notes in Appendix B). Here, only the normalized infected host proportion is shown; the sporozoite rate is absent. These trajectories are for hosts with any parasite density in their blood, *i.e.* those with 0.01 parasites/*μ*L or greater, and not for patent hosts. This standard has been chosen for a congruent comparison with the Ross/Macdonald model, where simply a host either harbors parasites or does not. Intervention coverages of 85% are used and the durations of both interventions are those specified above. No decay is specified for the IRS in the simulation, but it is configured generically as a step function, on during its effective period and then off. Trajectories are normalized by the average prevalence in this community absent interventions. Separate, otherwise equivalent, intervention-free simulations are run to determine these averages, which amount to a determination of X* (and Z* if desired), the prevalence balance points of Eq. (3). Stochastic noise is clearly evident in the eight trajectories plotted. Some of the simulated infected host prevalence trajectories shown in Fig. 5 are for a community absent any immune protection, and others with the *openmalaria* default for partial immunity [41]. Again, more details of the simulation procedure and the interventions can again be found in Appendix B. A first comparison of all simulations with the Ross/Macdonald variant is that the recovery times, post-intervention are *much longer* for the simulation. Eq. (12) indicates the community regains half of its infected proportion for *τ_m_* ≈ 2 ($R_{0}=25$), agreeing with the Ross/Macdonald trajectories of the plot. In contrast, the simulations here with immunity easily predict three times that. Furthermore, at nearly three months ($\bar{\tau }\approx 8$ post-intervention), the Ross/Macdonald theory indicates the effects of the MDA are essentially gone while it takes perhaps slightly more than a year for the equivalent equilibration in *openmalaria*. These restoration times, the hangover of these interventions, are substantially different. Malaria invades the community post-intervention with *openmalaria* at a much slower rate, with and without acquired immunity in the populace. Thus post-MDA, the left panel of the figure, shows one apparent role of acquired immunity in the simulations: it protects the community from the rapid return of widespread infections. A faster return to the proliferation of parasitemia is evident when immune protection is absent. It is, though, not as responsive as the simple Ross/Macdonald variant model presented above. Which situation better reflects a real (idealized) community suffering from invading malarial infections after a retracted intervention is to us, uncertain. As mentioned above, the Ross/Macdonald rates of Eq. (12) should be regarded as a fast upper limit for recovery due to the abrupt return to the pre-intervention force of infection (likely an overestimate of transmission at this point in time), and absent immunity. Partial, acquired immunity is instated for all further simulation results below, and the more ponderous return to equilibrium prevalence will be apparent.

The IRS deployed in the middle panel of Fig. 5 also presents an interesting comparison. First, IRS simulated by *openmalaria* is not as effective as IRS modeled by the Ross/Macdonald variant. As mentioned in Section 3.2 above, the rate at which host infections are lost (*r* in Eq. (2)) essentially determines the diminishing slope, or this effective rate of infection loss in $\bar{X}$ during the IRS, as only a trickle of new infections amend that rate. Here, in simulation, infections appear to either expire much more slowly, or more than a small number of new infections take place in the IRS period. As discussed in Appendix B, the IRS potency is maximized in this application, with pre- and post-prandial mortality affecting essentially all contacted mosquitoes. The heterogeneity of host selection, an included sophistication in the transmission model built into *openmalaria*, may also play a role in the weaker overall impact of the IRS.

As a comparison, in both left and center panels, it is intriguing that the rate infections are acquired by a community post-intervention is *slow* for *openmalaria*, which is in part due to effects of acquired immunity, but overall the effective force of infection is simply less potent. The combined MDA+IRS campaigns on the right indicates the same trend: the interventions indicate a synergy with joint deployment. Significant gains are again established with synchronous interventions. An inset in Fig. 5 demonstrates these gains, as measured by the impact of Eq. (20) for $R_{0}=25$. There is first a comparatively weak IRS, but more significantly, an impact that is *better than double* for synchronous deployments of a MDA and IRS. The *openmalaria* simulations demonstrate a synergy that is even larger than that of the Ross/Macdonald model. This is the same simple mechanism at work as seen above: the host reservoir is cleared by the MDA and the vector control prevents it from being refilled. Deployed separately, this coordination is clearly absent and the overall impact is diminished.

## 5 Discussion

Both MDA and IRS are potent transmission suppressing interventions, if performed with high population coverage. Given their complementary impacts on the human and vector parasite reservoirs, the uncovered synergy between these interventions is both logical and potentially valuable for malaria control programming. In order to explore the nature and size of this potential synergy, we have extended a simple Ross/Macdonald variant to incorporate these two interventions, MDA and IRS, and have carried out a concise analysis of their overall impacts on population infection.

Each intervention is first modeled in isolation, with intensities of either of the interventions set to the same $R_{0}^{I}$ through the duration of the campaigns, an attempt to put them on equal ground considering transmission. The factors of amplification: {*ξ, μ, κ*} are all introduced as accelerants which manually depress the reproductive number *R*_0_ during the interventions, and are all fairly logical: in an MDA *ξ* decreases the duration of host infections and *μ* provides a prophylactic effect, while *κ* is responsible for killing mosquitoes during an IRS campaign.

The MDA campaign cleanses the host parasites of 85% of the population and having a modest chemoprophylactic effect, protects the recipients for a short duration. After this, individuals are again susceptible and infections are re-established. The dynamics of the Ross/Macdonald model dictate that these infections restore an equilibrium, or a balance of parasite transactions, post-intervention. The timeline to this re-equilibration, that of relaxing to the stable equilibrium point of Eq. (3), is simply related to the force of infection of the entomological setting the system relapses into. High transmission has a fast relapse to the proliferation of parasitemia, a phenomenon explored in greater detail in Appendix A. This is an approximation, one that is perhaps too fast as an estimate, but one that enables us to discern the forces responsible for restoring the equilibrium, and how this rate scales with *R*_0_.

The IRS campaign impacts the vector, changing the effective entomological setting of the community. A simple population model for the mosquito population is incorporated which, when diminished, affects the effective $R_{0}^{I}$ of the intervention. The IRS, with its much longer duration set by the effective period of the insecticide, inhibits the transmission of parasites by the vector, and leaves hosts with (potentially) no other means of purging infections except to clear on their own slow timescale. Host infections/infectiousness wane very slowly but the populace has protection from new infections through the reduced vector population.

It is sensible that a strategy with combined campaigns would have more impact than isolated campaigns. If the IRS can only offer the protection of reduced biting, while leaving individuals to fight infections off on their own, it is clear that a simultaneously deployed MDA offers the additional, and otherwise absent, therapeutic aspect. The analysis above underlines that a clearing of the host reservoir with an MDA but aided with the added protection of the IRS is a good strategy; the MDA clears infections while IRS effectively protects the cleared individuals from reinfection, adding endurance to these initial gains. The *openmalaria* simulations confirm this effect and its size are not specific to the Ross/Macdonald model. Lastly, the impact of synergy here is measured in terms of its effect on host-prevalence alone, in terms of those infections (of any parasite load) averted with interventions. An analysis of clinical incidence of disease, instead of the impact on prevalence, would introduce a layer beyond the simple analysis presented here. This synergy, with its extremely simple mechanism, is here revealed through its impact on prevalence alone; a standard metric of malaria in a community.

Simulations were carried out using intentionally simplified settings in order to unmask the fundamental transmission dynamics, and do not necessarily represent a real community or reflect a specific entomological environment. For example, vector biting in our simulations has no seasonal oscillations, and is carried out by a single species. There is also very weak case management. More sophisticated (and real) environments could be simulated but these embellishments might obscure the result (case management, for example, may be represented through a health system parameterization but functions as an additional “background” intervention). The Ross/Macdonald model employs the most basic of transmission dynamics, contains essentially no elaborations, has very few parameters and indicates a strong synergy, together with the full transparency of the forces and effects that enable it. The more complex *openmalaria* simulation, incorporating a much broader picture of transmission and the many pertinent forces that shape it, confirms this synergy, and in fact predicts a comparable, if not greater impact. Our intent has been to eludicate generalities that are scaling-level trends and, we hope and expect, are *model-independent* by nature. We anticipate other transmission models would echo substantively similar results.

## 6 Conclusion

We have considered the isolated and combined impacts of two interventions, MDA and IRS, and through two disparate modeling efforts shown the community effects of these interventions is greatly enhanced by their combined application in the setting. This synergy is found to be robust both by the semi-analytic Ross/Macdonald variant and *openmalaria*. The mechanism of this cooperative impact is illustrated by the semi-analytic model, and also readily understood in terms of basic transmission dynamics.

Additionally, we have also presented a scaling relationship that shows how quickly malaria infections are re-established following an intervention in a simple scenario absent any immunity effects. Not only is resurgence in a strong transmission environment swifter than resurgence in a weak transmission environment, scaling with $R_{0}^{-1},$ resurgence is shown to be slower for more effective interventions. In the simple scenarios we are modeling, it is plainly beneficial to permanently alter the entomological environment in order to prevent resurgence: this is an obvious result, but one that also highlights the need for integrated vector management.

## Figures and Tables

**Fig. A1:**
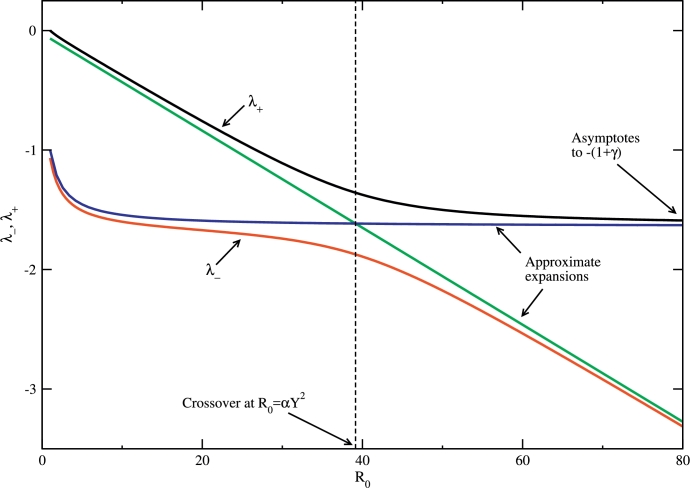
The eigenvalues of Eq. (A.2) as a function of *R*_0_, with the asymptotic expansions of Eq. (A.4)–(A.5). The crossover region referenced in the text is also indicated. The parameters *γ* and *α* are as before.

## Appendix A Details of the stability analysis

Eqs. (1)and (2) describe a simple first order, autonomous nonlinear set of equations, and they mimic predator/prey models. Combined with initial conditions, $X\left(t=t_{0}\right)=X_{0}$ and $Z\left(t=t_{0}\right)=Z_{0},$ it constitutes (at least in principle) a solvable system with a unique trajectory. Dynamical stability is assessed from a Jacobian matrix whose eigenvalues categorize linearized trajectory behavior. For the system of Eqs. (5) and (6), it is written,

(A.1) $$J\left(\bar{X},\bar{Z}\right)=\left[\begin{array}{cc} -\left(\alpha +\beta Z^{*}\bar{Z}\right) & \beta Z^{*}\left(\frac{1}{X^{*}}-\bar{X}\right)\\ \gamma X^{*}\left(\frac{P_{e}}{Z^{*}}-\bar{Z}\right) & -\left(1+\gamma X^{*}\bar{X}\right)\; \end{array}\right],$$

which, evaluated at the trivial, elimination point $\bar{X}=\bar{Z}=0$ has a larger eigenvalue that determines stability (the other is always negative). The larger is positive for $\gamma \beta P_{e}-\alpha >0,$ or *R*_0_ > 1 as is well known. All trajectories for reproductive numbers *R*_0_ < 1 thus attract to the elimination point and all others greater than unity are unstable with respect to elimination. As long as *R*_0_ < 1 is maintained, elimination is the only long-term outcome for system evolution. The analysis below is an extension of work pioneered by Lotka [49] and other, comparable analyses have been performed by other authors [50], [51].

The eigenvalues of the Jacobian for the second equilibrium point {*X**, *Z**} may be written,

(A.2) $$\lambda_{\pm }=-\frac{1}{2}\left(\frac{R_{0}}{Y}+\alpha Y\right)\pm \sqrt{\frac{1}{4}{\left(\frac{R_{0}}{Y}-\alpha Y\right)}^{2}+\alpha },$$

with

(A.3) $$Y=\frac{R_{0}+\gamma }{1+\gamma }.$$

which shows the discriminant is always positive. Eigenvalues are thus never imaginary and as such there are no cyclic, *i.e.* periodic solutions common to some predator/prey systems. All trajectories instead exist in attraction basins and terminate at equilibrium points, or potentially run off to infinity. Further inspection indicates that the latter is only possible for a negative determinant, which upon simplification is only the case for *R*_0_ < 1, precisely the region stable for elimination. Thus, the elimination point $X^{*}=0,$ $Z^{*}=0$ and the nontrivial one given in Eq. (3) are both stable fixed points and as such the system asymptotes to either, based on *R*_0_ alone.

The quantities *αY* and *R*_0_/*Y* are nondimensional “bare” rates at which infections are acquired near *X**, *Z** for humans and mosquitoes. Closer examination of Eq. (A.2) shows that the eigenvalues have a crossover region when *R*_0_/*Y* ≈ *αY*, which corresponds to a curious symmetry point where these parasite acquisition rates are nearly the same in both hosts and mosquitoes. Pre-crossover, expanding for small *αY*^2^/*R*_0_, these rates are,

(A.4) $$\lambda_{+}\approx -\alpha Y=-\frac{\alpha R_{0}}{1+\gamma }-\frac{\alpha \gamma }{1+\gamma },$$

and

(A.5) $$\lambda_{-}\approx -R_{0}/Y=-\frac{R_{0}\left(1+\gamma \right)}{R_{0}+\gamma }.$$

Beyond this crossover, for large *R*_0_, the rates *swap*. In this limit, expanding Eq. (A.2) about small *R*_0_/*αY*^2^, $\lambda_{+}\approx -R_{0}/Y$ and $\lambda_{-}\approx -\alpha Y$. These expansions, the crossover, and the viability of these expansions can all be seen in Fig. A.6. Again, these expressions swap regions of applicability with the crossover, *i.e.* $\lambda_{+}↔\lambda_{-},$ so that the approximation for $\lambda_{+}$ pre-crossover is an adequate approximation for $\lambda_{-}$ post-crossover.

These eigenvalues are the non-dimensional rates, in units of *g*, at which the infectious densities regain equilibrium values. To a good approximation (for small *α, i.e.* momentarily neglecting the small cure rate), host infections are acquired with the elemental solution, $\exp(\lambda_{+}\tau )$ with $\lambda_{+}$ given by Eq. (A.4) and (A.5) pre- and post-crossover, respectively. In the pre-crossover (low and modest *R*_0_) regime, host infectiousness rebounds with a rate dominated by *R*_0_,

(A.6) $$\lambda_{+}=-\alpha \frac{R_{0}+\gamma }{1+\gamma }\approx -\frac{\alpha R_{0}}{1+\gamma }.$$

The constant term in Eq. (A.4) is dwarfed for modest *R*_0_ and dropped. Eq. (A.6) indicates the sensible result that a larger *R*_0_ has a faster recovery. In contrast, post-crossover with large *R*_0_, the rate saturates to that of $\lambda_{-}$ above and asymptotes to $\lambda_{-}\to 1+\gamma$ (see Fig. A.6) which is curiously *R*_0_ independent. There is a limit to how fast equilibrium can be recovered—an inertia of the vector reservoir—present even in large *R*_0_, which is a limiting rate due to the ephemeral lifecycle of the vector. It is noteworthy that pre-crossover this rate *grows* with *R*_0_ and then post-crossover, *saturates*. Even for the highest transmission settings, the rate at which equilibirum is recovered never exceeds 1+γ.

### A1 Approximating the time to re-equilibration

In the linearized approximation, post-intervention the system decays to the equilibrium points of Eq. (3) with the general solutions $\exp(\lambda_{+}\tau )$ and $\exp(\lambda_{-}\tau ),$ which (somewhat unfortunately) tend to be accurate for small perturbations from the equilibrium. As the heavy suppression of infections with an MDA campaign or the loss of infectious vectors with an IRS are neither small dynamical effects, recovery tracked with this exponential decay is a poor estimate.

One reasonable approximation to estimate recovery trajectories is to exploit the fact that the sporozoite rate $\bar{Z}\left(\tau \right)$ responds quickly to changes in the host reservoir of $\bar{X}\left(\tau \right),$ and thus all recovery trajectories in $\bar{Z}\left(\tau \right)$ in Fig. 1, Fig. 2, Fig. 3 track $\bar{X}\left(\tau \right)$ very well. Denoting the host infection proportion at intervention expiry as ${\bar{X}}_{f}=\bar{X}\left(\tau_{0}+\bar{\tau }\right)$ and simply equating $\bar{X}\left(\tau \right)=\bar{Z}\left(\tau \right)$ (which is naturally best when they are reasonably close as in Fig. 3), Eq. (5) simplifies to,

(A.7) $$\begin{array}{ccc} \frac{d\bar{X}}{d\tau } & = & \beta \left(\frac{Z^{*}}{X^{*}}\right)\bar{X}\left(1-X^{*}\bar{X}\right)-\alpha \bar{X} \end{array}$$

(A.8) $$\begin{array}{ccc} & = & {\bar{f}}_{I}\bar{X}\left(1-\bar{X}\right). \end{array}$$

The rate prefactor ${\bar{f}}_{I}=g^{-1}f_{I}\left(X^{*}\right)\approx \lambda_{+}$ appears without further approximation, and is the ambient non-dimensional force of infection mentioned in the text (Eqs. (10) and (11)). It is that of the entomological setting the system relaxes into. This governing equation is valid for pre-crossover dynamics, and the substitution, $\lambda_{+}↔\lambda_{-},$ approximates the solution post-crossover.

Eq. (A.8) is easily integrable, with solution,

(A.9) $$\bar{X}\left(\tau \right)=\frac{1}{1+n_{0}e^{-{\bar{f}}_{I}\tau }},\;\;\text{with},\;\;n_{0}=\frac{1-{\bar{X}}_{f}}{{\bar{X}}_{f}},$$

and *n*_0_ an obvious integration constant determined by the initial condition $\bar{X}\left(\tau =0\right)={\bar{X}}_{f},$ the infectious host density at intervention expiry. Here, the assignment τ=0 corresponds with the moment these dynamics begin, namely the instant the effective period of the intervention concludes.

Defining a characteristic recovery time as that to regain half of equilibrium, (as is typical), an estimation for the time the community re-acquires malarial infections is,

(A.10) $$\tau_{m}=\frac{1}{{\bar{f}}_{I}}\log\left(\frac{1+{\bar{X}}_{f}}{{\bar{X}}_{f}}\right)\approx \frac{1+\gamma }{\alpha R_{0}}k_{f}=k_{f}\tau_{+},$$

which is, again, measured in units of the average mosquito lifetime $g^{-1}$. The final equality defines the unitless number *k_f_* which is multiple, *k_f_* ≲ 5 based on the depth of infection suppression accomplished by the campaign. The recovery time is found to grow substantially with the depth of suppression of the host infectivity, ${\bar{X}}_{f}$. Thus, the more effective an intervention is, *i.e.* squelching ${\bar{X}}_{f},$ the *longer* the community takes to rebound back to equilibrium (Eq. (3)) with prevalent parasitemia. The recovery time is essentially the inverse force of infection stretched by $k_{f}=\log\left(1+{\bar{X}}_{f}^{-1}\right)$ (which, for low coverage, is a reduction).

The advantage of this analysis of the nonlinear system is twofold. First, the pre-crossover restoration rate is established to be exactly the ambient, equilibrium force of infection for the community pre-intervention, ${\bar{f}}_{I},$ an entirely sensible result since *R*_0_ is immediately established post-intervention as a simplification. This recovery rate also confirms the stability analysis, since for *R*_0_ > 1,

(A.11) $${\bar{f}}_{I}=\alpha \frac{R_{0}-1}{1+\gamma }\approx \alpha \frac{R_{0}}{1+\gamma }=-\lambda_{+},$$

the leading term of the relaxation rate above is recovered. Second, the form of regaining equilibrium, Eq. (A.9), yields a characteristic time *τ_m_* which is an estimate for the reacquisition of malarial infections in the community. It is dependent on both the reproductive number *R*_0_ and the net effectiveness of the campaign, information contained in ${\bar{X}}_{f}$. For a campaign such as that of the combined MDA+IRS in Fig. 3 (top), with $R_{0}=12.5,$ from Eq. (A.10) it takes *τ_m_* ≈ 6 (6 average mosquito lifecycles, perhaps 2 months) to regain half-again the infection prevalence after intervention expiry. Furthermore all intervention effects are essentially gone a bit more than half a year post-retraction. Parameters for this estimate are those in the caption of Fig. 1. In this case, the combined interventions suppress host infections to below 5% and anopheles infections to about 1%. After just 6 months, infections and infectiousness re-establish to roughly pre-intervention levels, which according to Eq. (3), approaches *X** ≈ 0.875 for human hosts and *Z** ≈ 0.13 for anopheles.

## Appendix B Details of the *openmalaria* simulations

All simulations were run with the base model, which is often referred to as R0001 [40], [47] and simulated with version 33. The background and details of building and the determination of the integral 31 parameters which constitute the transmission model are detailed elsewhere [40], [41], [42], [43], [44], [45], [46], [47], [52]. Transmission dynamics are discretized into 5-day timesteps for infectious/infectiousness status updates in hosts and mosquitoes, extrapolated parasite densities, forecasted acquired immunity, and severity of malarial episodes. Clearly the dynamics of immunity are neglected in the simple Ross/Macdonald variant considered in the text though it is included in simulations here [41]. Many different dynamical elements for transmission are considered in *openmalaria* which separate these simulations from the semi-analytic model in the text: superinfection is included, acquired partial immunity in a few different flavors is built-in (as designed, immunity may be a result of the number of prior episodes or a prior high burden of parasite densities, or due to maternal protection), demography, and health systems access and effects.

Separately the entomology and dynamics of parasite transmission is specified through a time-lagged deterministic difference equation which accounts for heterogeneous infection of hosts [53], [54]. Transmission may also be seasonal though it is here forced to be constant to afford a simple and direct comparison. An additional 16 parameters specify the life and feeding cycle of the vector, which is taken to be those of a single vector species, *anopheles gambiae*, whose parameters are chosen as previously specified [55] (in an included supplement). The one exception to these parameter assignments is that the extrinsic incubation period is set to n=10 days in accordance with the average lifetime $g^{-1}$ mentioned in the text (which sets $P_{e}=e^{-gn}=e^{-1},$ also, for comparison it is n=11d in the reference). A correspondence between the EIR E and the reproductive number *R*_0_ is needed to align the simulations with the semi-analytic model. Ref.[48] details different effective *R*_0_ values for *openmalaria* model simulations, with varying treatment probabilities. For the case of the base model without case management, the reproductive number very nearly corresponds with the annual EIR (total bites per host per annum) $E_{a}$=365dE. To this end, simulations were run with annual EIR values equal to the reproductive number.

A health system is somewhat generically parameterized, as detailed in the *openmalaria* wiki pages [56]. Defaults for simulations where case management is not a primary interest of the simulation are used, including those for access, adherence, and compliance. Uncomplicated cases of malaria are treated for only 4% of the populace and for 48% of severe episodes. Cure rates are unity for those who access care. This very low level of care allows reasonable correspondence with cases of ref.[48]. Other case fatality rates are as is typical as well as specified sequelae rates.

Finally, interventions are modeled as closely as possible with those described in the text in the semi-analytic model. The effects of the IRS do not slowly decay but are on or off through the use of a step function which activates the intervention from deployment for a duration of 36 timesteps, or 18$g^{-1}$. The IRS is modeled and deployed as a GVI (generic vector intervention) and *maximized* in its mosquito killing impact, with preprandial and postprandial killing effects set to 0.99; deterrency is set to zero. The MDA is modeled as clearing the liver for 5 days and blood for 4 timesteps, or 2$g^{-1}$. Coverage is 85% for both interventions, corresponding to the text. Since about 15 days are required for a full response in the sporozoite rate with vector control in *openmalaria*, to compare the timing of interventions, the time index of intervention deployment is shifted for an apt comparison. This means, for the Ross/Macdonald variant model, an IRS deployed at *τ*_0_ has the sporozoite rate instantaneously reduced at this moment. An equivalent *openmalaria* deployment is then begun at *τ*_0_-(15d/10d), since this accounts for a latent response in the sporozoite rate [53]. We take, for reasons of simplicity, the timing of an IRS campaign as defined to be the very moment that the sporozoite rate plummets. On this note, the MDA timing is also shifted. An MDA deployed within *openmalaria* at time $t_{0}=k\Delta t$ has its first effects at timestep k+1, and thus comparisons are made for a MDA deployed at *τ*_0_-(5d/10d) with a Ross/Macdonald campaign deployed at *τ*_0_.

Simulations are performed on a population of N=10000 individuals with a demographic of that of Ifakara, as detailed in reference [41]. As none of the analytic results in the text above depend on this demography, the distribution is rather irrelevant in this context, but necessary as the force of infection is dependent on an age distribution in *openmalaria*. The distribution was selected for ease of reproducibility and generality, and it also is representative of the general youthfulness of many sub-Saharan populations. The choice of population size is not selected with a community in mind but rather to simulate a large enough populace to have statistical relevance with small fractional random error. The multiple simulation results plotted demonstrate the stochastic variance in trajectories.

One complication with *openmalaria* and measuring the impacts of interventions is that in higher transmission settings, high-impact interventions will cause a resurgence in parasitemia that rebounds and can exceed the previous, pre-campaign equilibrium. The impact measure *I* of Eq. (20) breaks down in these circumstances. As can be seen in Fig. 5, this is not the case here, largely due to the relatively brief interventions. This does not mean that the impact measure *I* is trouble-free in that post-intervention the long-time limit for integration must be set. With the simulated $\bar{X}\left(\tau \right)$ equilibrium not being exactly that of pre-intervention, the noisy post-intervention trajectory likely bounces around a nearby equilibrium. To this end, we set the impact integration window to roughly 8 years post-intervention. This means we cut off effects of noise  ∼ 8 years (580 timesteps) post-intervention, and this is certainly long after these campaigns. Larger windows add greater dispersal about the values of the impact in Fig. 5, as more noise contributes to the sum, but the magnitude of the absolute impact is essentially conserved.

Lastly, cases without immunity are run for 80 years without immune protection and interventions are deployed in the last 5 years of this time window. This is essential to equilibrate the system without the added protection of partial, acquired immunity.
